# Testosterone levels and symptoms of hypogonadism in Swedish men: a prospective study from the Vara – Skövde cohort

**DOI:** 10.3389/fendo.2026.1882696

**Published:** 2026-07-17

**Authors:** Amar Osmancevic, Ying Li, Kristin Ottarsdottir, Margareta Hellgren, Ulf Lindblad, Matthew Allison, Penelope Trimpou, Spela Kokelj, Bledar Daka

**Affiliations:** 1 General Practice/Family Medicine, School of Public Health and Community Medicine, Institute of Medicine, Sahlgrenska Academy, University of Gothenburg, Gothenburg, Sweden; 2 Division of Preventive Medicine, School of Medicine, UC San Diego, San Diego, CA, United States; 3 Department of Endocrinology, Institute of Medicine, Sahlgrenska Academy, University of Gothenburg and Sahlgrenska University Hospital, Gothenburg, Sweden; 4 Sahlgrenska University Hospital’s Research, Education, Development & Innovation Unit, Sahlgrenska University Hospital, Gothenburg, Sweden

**Keywords:** aging, endocrinology, epidemiology, hypogonadism, men, testosterone

## Abstract

**Background:**

Debate persists regarding the diagnosis of late-onset hypogonadism and the symptoms attributed to low testosterone.

**Aim:**

To examine cross-sectional and longitudinal associations between endogenous testosterone levels and hypogonadal symptoms in community-dwelling men, and to explore whether these associations vary by immunoassay method.

**Methods:**

We analyzed 657 men from the Vara–Skövde cohort who provided blood samples at baseline (2002–2005) and follow-up (2012–2014), completing the Aging Male Symptom questionnaire at the second visit. Endogenous *total* testosterone was measured by two different immunoassays. Given the systematically elevated *total* testosterone concentrations observed at the second visit, we recalculated to compare appropriately between visits. Associations were evaluated with linear and ordinal logistic regression models. Multivariate regression models were performed in four models to account for potential confounding variables.

**Results:**

In cross-sectional analyses, men with low *total* testosterone had higher scores than those with normal testosterone concentration in the sexual domain (final model; Mean difference = 1.7; 95% CI: 0.4, 2.9; p = 0.01). No differences were observed between intermediate and normal testosterone categories. Moreover, while directly measured testosterone values at second visit yielded a lower prevalence of biochemical hypogonadism compared to recalculated *total* testosterone levels, the sexual symptom burden remained consistent. Testosterone levels at baseline were not associated with *total* AMS score in men after ten years.

**Conclusion:**

In cross-section, low endogenous *total* testosterone concentration was associated with higher sexual symptom burden in men. While measurement platforms showed numerical shifts in testosterone values, the clinical association with symptom burden remained consistent, with the modern assay identifying the most symptomatic individuals.

## Introduction

In healthy men, testosterone, the principal male sex hormone, shows a slow, progressive age-related decline after age 50, of which most do not reach biochemical hypogonadism (less than 8 nmol/L). An even steeper decline is observed in levels of bioactive fractions (1–3). This is more pronounced in younger aged men with chronic diseases such as obesity, type 2 diabetes (T2DM), or metabolic syndrome, as well as those receiving certain medications (2, 4–6). Moreover, while data remains discrepant, studies suggest that men with lower baseline testosterone tend to experience a steeper decline over time than eugonadal men (7, 8). When accompanied by clinical symptoms, this condition is commonly referred to as late-onset hypogonadism (4, 9). However, considerable debate persists regarding the diagnosis of hypogonadism, the spectrum of symptoms attributable to testosterone deficiency, and appropriate treatment strategies.

In recent decades, several questionnaires have been developed to identify men with potential symptomatic testosterone deficiency (10). However, studies generally report only weak correlations between reported symptoms and biochemical testosterone levels. Despite this, there has been a marked increase in both the diagnosis of testosterone deficiency and the prescription of testosterone treatment. For example, a recent study from Sweden documented an almost six-fold rise in the prevalence of hypogonadism among men aged 45–64 years, accompanied by a 750% increase in testosterone prescriptions over the last two decades (11).

Accordingly, the aim of this study is to examine the association between *total* and calculated *bioavailable* and *free* testosterone with symptoms potentially related to late-onset hypogonadism in men, as measured by the Aging Male Symptom (AMS) score. A secondary aim is to assess whether the association between testosterone concentrations and AMS scores varies by assay method.

## Methods

### Study design and study population

A random sample from the population census register was used to establish the Vara-Skövde cohort a longitudinal study conducted in southwestern Sweden between 2002 and 2005 with a follow-up visit between 2012 and 2014 (12). Briefly, the cohort originally enrolled a random population sample of 2,814 individuals aged 30–74 years (including 1,400 men), with oversampling of participants aged 30–50 years. This strategic sample size was informed by extensive experience from previous studies addressing similar research questions in the Skaraborg region and previously received highly favorable evaluations from the Swedish Medical Research Council (MFR). The study included participants from both rural and urban settings in southwestern Sweden, the vast majority of whom were Swedish-born. Vara represented a small rural community, whereas Skövde represented a larger urban community. From this original group, a representative subsample of 944 men was invited to participate in a follow-up visit of which 657 men completed protocols that were identical to those used at baseline with the exception of the AMS questionnaire which was introduced at second visit upon expert endocrinological consultation (Dr. Thord Rosén) to investigate age-related clinical symptomatology as the cohort matured. All men with missing data at both baseline and follow-up, including incomplete responses to the Aging Male Symptoms (AMS) questionnaire or missing information on lifestyle factors, comorbidities, blood samples, were excluded from the analyses.

The primary aim of the Vara-Skövde study was to investigate the progression from normal health through preclinical stages to overt disease, with a particular emphasis on sex differences in cardiometabolic risk.

### Assessment of hypogonadal symptoms

Symptoms related to hypogonadism were assessed at the second visit using the Aging Male Symptoms (AMS) questionnaire (13). This scale was developed to measure symptoms in aging men that could potentially be related to hormonal changes, similar to menopause rating in women. The questionnaire comprises 17 questions grouped into three symptom clusters: psychological (5 questions), somatovegetative (7 questions), and sexual (5 questions). Each question is scored on a scale from 1 to 5, corresponding to symptom severity: 1 = none, 2 = mild, 3 = moderate, 4 = severe, and 5 = extremely severe. Men were further categorized into asymptomatic (less than 26 points), mild (27–36 points), moderate (37–49 points) and severe (50 points or above) (13).

### Assessment of endogenous sex hormones

Morning samples were collected following an overnight fast, promptly processed, and stored at −82 °C until analysis. Serum testosterone concentrations at baseline were determined using the Access Testosterone Assay (Beckman-Coulter, catalog no. 386982A, RRID: AB_2895595), with a reported coefficient of variation (CV) between 7% and 8%. During follow-up, testosterone was analyzed using the Elecsys Testosterone II Assay from Roche Diagnostics (catalog no. 05200067, RRID: AB_2783736), demonstrating improved precision (intra-assay CV: 1.6–2.6%; inter-assay CV: 2.3–5.1%). The limit of quantification was set at 1 nmol/L for both assays. *Bioavailable* and *free* testosterone concentrations were calculated using the established formula by Vermeulen et al. (14).

Due to methodological changes over the study period, assay comparability was evaluated by analyzing testosterone concentrations within similar age categories at baseline and follow-up. This revealed that testosterone measurements obtained at second visit were consistently 11% higher compared to baseline values. Accordingly, prior evaluations have shown that this assay systematically overestimates measured values compared to liquid chromatography mass spectrometry (LC-MS/MS) (15). To assess whether this difference influenced the associations, sensitivity analyses of *total* testosterone at the second visit were conducted using both the adjusted calculated values (multiplied by a factor of 0.89) and the directly measured values (12). Sex hormone-binding globulin (SHBG) were assessed using radioimmunoassay (Siemens IMMULITE 2000XPi; coefficient of variation 5%).

**Figure 1:**
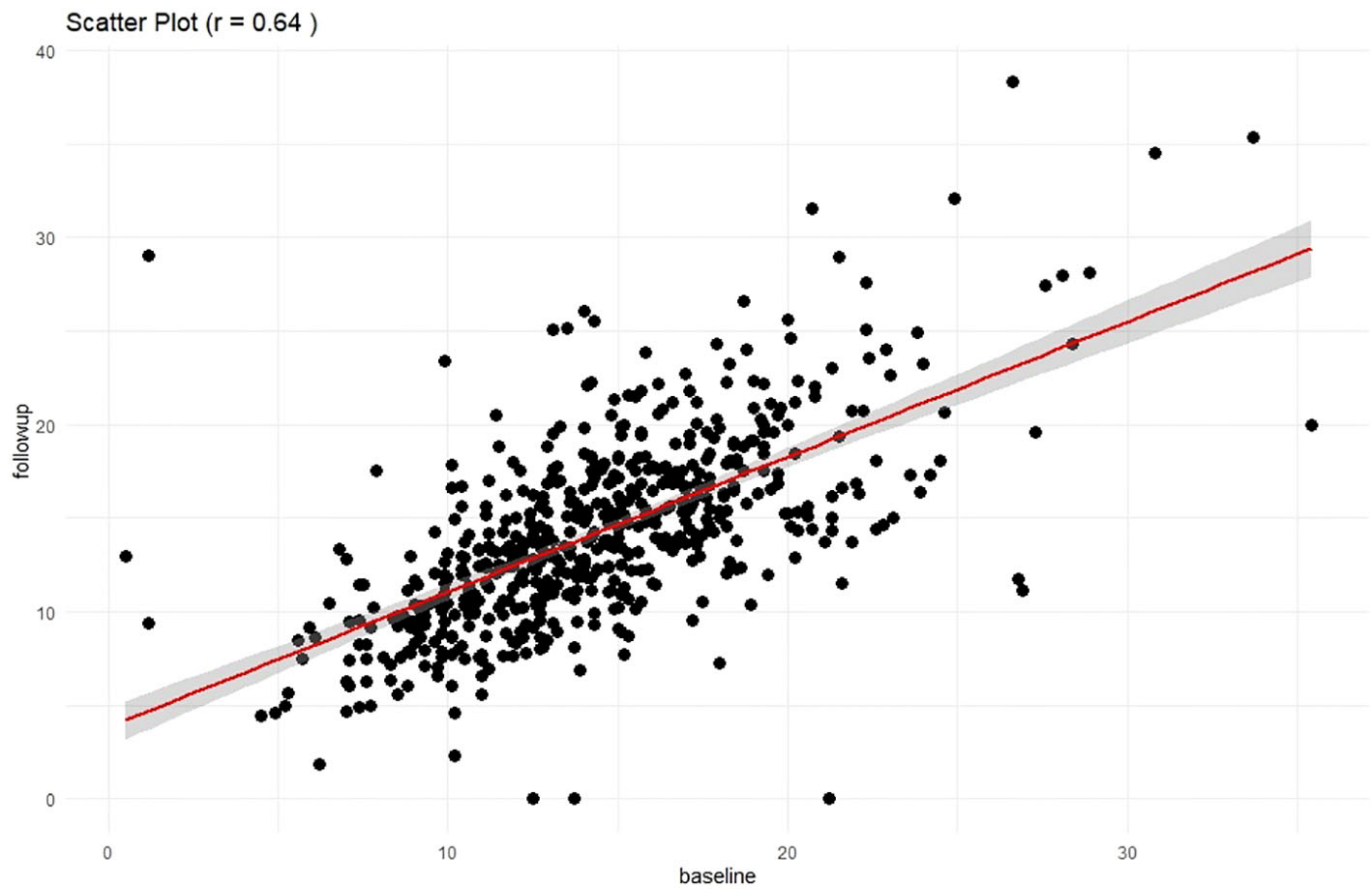
Longitudinal correlation of total testosterone levels between first and second visit. The scatter plot demonstrates individual tracking and longitudinal stability of total testosterone concentrations over the 10-year study interval (r=0.64).

A moderate correlation between baseline and follow-up testosterone concentrations was confirmed (Pearson correlation: r = 0.64, p < 0.001) (**Figure 1**). Assay completion was 95% at both visits.

### Data collection and covariates

Experienced study nurses systematically gathered detailed information regarding participants’ medical histories, lifestyle factors and ongoing medication use. Additionally, comprehensive anthropometric measurements and blood pressure assessments were carried out. Waist-to-hip ratio (WHR) was calculated as waist circumference divided by hip circumference. Blood pressure was consistently measured in a supine position following a resting period of five minutes, adhering to standardized protocols at both baseline and follow-up examinations. Leisure-time physical activity was assessed through participants’ responses to a single question regarding their typical weekly activity level during leisure hours during the past year (16). Participants selected one of four possible responses: (1) predominantly inactive, such as reading or watching television; (2) lightly active, involving at least four hours per week of activities like walking, cycling, gardening, or commuting by foot or bicycle; (3) moderately active, including at least two hours per week of recreational activities such as jogging, swimming, or tennis; or (4) vigorously active, involving frequent engagement in physically demanding activities like jogging, swimming, or tennis several times per week. The questionnaire has been validated with earlier studies (17). Smoking status (current daily smoker: yes/no) and alcohol consumption were assessed by questionnaire. Weekly alcohol intake (g/week) was calculated from reported frequency and typical quantity of beer, wine, and spirits consumed over the past 30 days (18).

Clinical diagnoses such as type 2 diabetes mellitus (T2DM) and hypertension (≥ 140/90 mm/Hg) were defined in accordance with guidelines from the World Health Organization (WHO) for diabetes and the Sevent Report of the Joint National Committee (JNC-7) (19, 20). Hyperlipidemia was defined as self-reported or taking antihyperlipidemic medication. Additionally, high-sensitivity CRP (hsCRP) levels were analyzed at the Department of Clinical Chemistry, Skåne University Hospital, using RIA. The assay performance was characterized by intra- and inter-assay CVs of 1.8–6.2% and 2.8–11%, respectively (12).

### Statistical analyses

Descriptive statistics were used to characterize the study population at visit 1 and visit 2. Both cross-sectional and longitudinal analyses were performed to evaluate the associations between testosterone (*total* and calculated *bioavailable*) concentrations at first and second visit, with AMS scores assessed at second visit. Linear regression models were employed to examine associations between exposure and outcome variables. In line with previous studies and clinical guidelines, *total* testosterone concentrations were categorized as follows: <8 nmol/L (biochemical hypogonadism), 8–12 nmol/L (intermediate levels), and > 12 nmol/L (considered normal) (4, 21) Analyses were also done to assess continuous changes in testosterone (*total* and calculated *bioavailable*) levels through time. In addition, proportional odds logistic regression was used to evaluate relationships when outcome variables were categorized according to symptom score. AMS scores were classified based on symptom severity and subdivided into three distinct domains: sexual, somatovegetative, and psychological symptom clusters to evaluate potential associations. If significant associations were identified after final adjustment, additional analyses were conducted to explore potential relationships between testosterone levels and individual symptom items within the AMS questionnaire.

Statistical models were adjusted stepwise across four theoretical models to account for potential confounding factors. Model 1 included unadjusted associations. Model 2 adjusted for age and waist-to-hip ratio. In our multivariable models, WHR was treated primarily as a confounder rather than a mediator. Specifically, visceral adiposity exhibits a complex, bidirectional relationship with the endocrine system, while it directly contributes to lower testosterone levels via increased aromatase activity and chronic low-grade inflammation (22), it also independently drives a wide range of symptoms (23), measured partly by the AMS questionnaire. Therefore, controlling for WHR was deemed methodologically necessary to isolate the independent association between testosterone levels and symptom burden, avoiding the overestimation of effect sizes that would occur due to residual metabolic confounding. Model 3 incorporated additional adjustments for sex hormone-binding globulin (SHBG), C-reactive protein (CRP), leisure-time physical activity (LTPA), smoking status, and alcohol consumption. Finally, Model 4 included further adjustments for the presence of type 2 diabetes mellitus (T2DM), hypertension, and hyperlipidemia.

Clinical guidelines suggest that men with intermediate *total* testosterone levels (8–12 nmol/L) but low *free* testosterone (<220 pmol/L) may be diagnosed with late-onset hypogonadism (LOH) (4). Therefore, we examined whether symptom burden differed between men with low versus intermediate versus normal *total* testosterone and either normal or low *free* testosterone concentrations. Furthermore, we performed sensitivity analyses to compare baseline characteristics between participants with complete versus missing AMS data, as well as between those who attended the follow-up visit and those who did not; these results are provided in the **Supplementary Tables**. All statistical analyses were performed using R software. A two-sided p-value <0.05 was considered statistically significant.

### Ethical considerations

Written informed consent was obtained from all participants prior to inclusion in the study and study protocol was approved by the Regional Ethical Review Board in Gothenburg, Sweden (D-nr Ö 199–01 and 036-12).

## Results

### Participant characteristics

**Figure 2:**
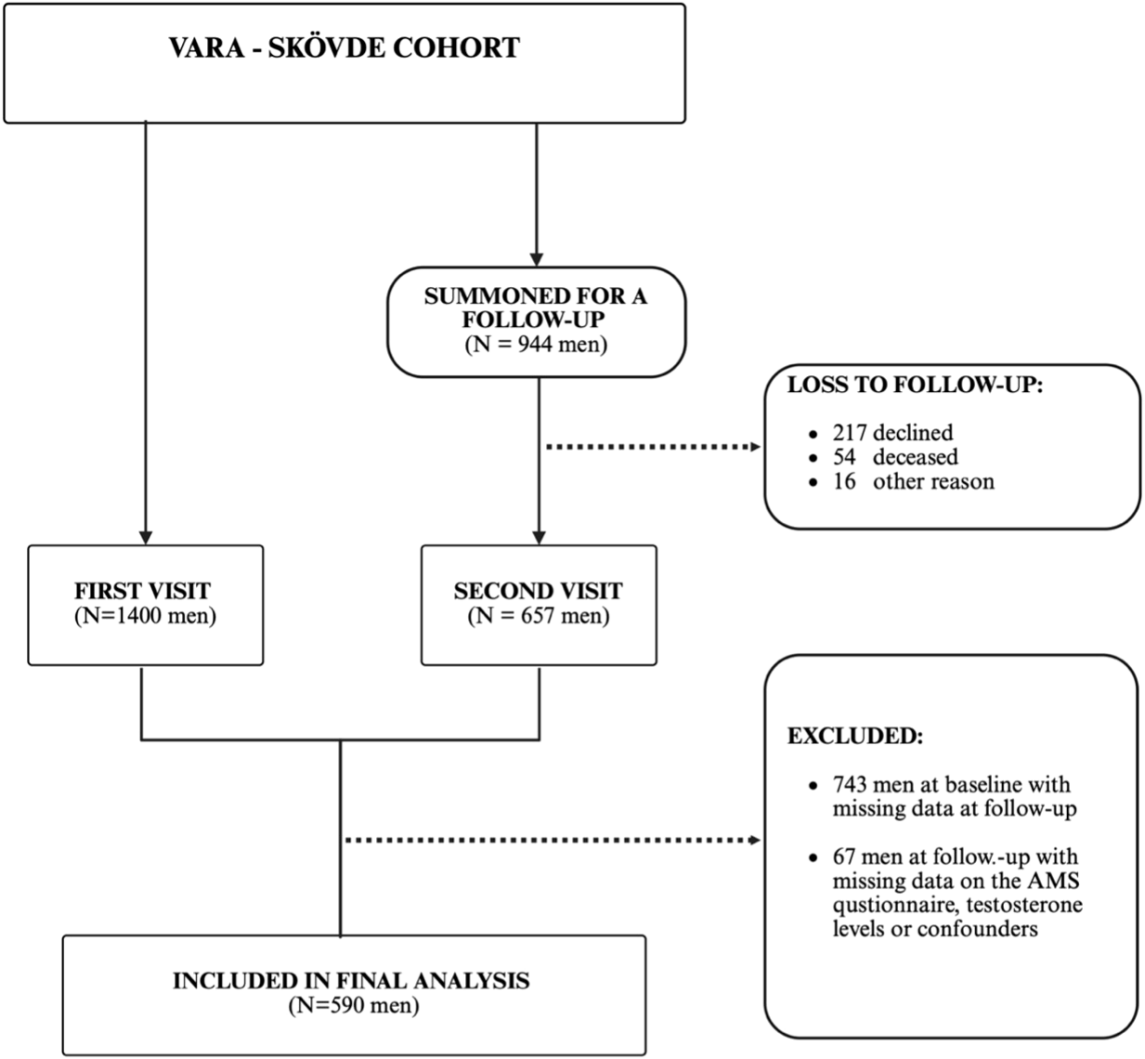
Flow chart of the study population.

The study population included all men who participated at baseline (n = 1,400) and were eligible for the second visit. Of these, 944 men were invited for re-examination. A total of 289 individuals were lost to follow-up due to non-response, death, or other reasons, resulting in 657 men attending the second visit. Among these, 67 were excluded due to missing data on key exposure, outcome, or confounding variables, yielding a final analytical sample of 590 men (**Figure 2**).

**Table 1** summarizes the baseline and follow-up characteristics of the study population. The median age increased from 47 to 57 years over the study period. WHR, BMI and alcohol consumption remained stable. Uncalibrated *total* testosterone levels increased (14.4 to 15.8 nmol/L) while recalculated *total* testosterone at second visit showed a modest decline (from 14.4 to 13.7 nmol/L). The number of men with biochemical hypogonadism (*total* testosterone < 8 nmol/L) was 32 at the first visit and 30 at the second visit. After recalculation, 52 men were classified with biochemical hypogonadism. The median AMS score at second visit was 29 points. The mean total AMS scores remained similar in men with *total* testosterone below 8 nmol/L at second visit (directly measured mean score 34 (± 11) vs. recalculated mean score 32 (± 11) (**Supplementary Table 1**). Additionally, cigarette smoking prevalence decreased, whereas the prevalence of T2DM, hypertension, hyperlipidemia, and self-reported vigorous physical activity increased.

**Table 1:** Characteristics of the study population.

Men (N = 590)	Baseline Median ± IQR//N (%)	Follow-up MeDIAn ± IQR//N (%)
Age (years)	49 (39 - 57)	57 (49 – 69)
WHR	0.94 (0.90 – 0.98)	0.97 (0.92 – 1.0)
BMI (kg/m^2^)	26.4 (24.5 – 28.8)	27.2 (25.0 – 29.3)
TT (nmol/L) > 12 8 – 12 < 8 (Recalculated TT< 8)	14.4 (11.3 – 17.1) 402 (68 %) 142 (26.6%) 32 (5.4%) N/A	15.8 (11.9 – 19.0) 443 (75%) 117 (20%) 30 (5.1 %) 52 (8.8%)
fT (pmol/L)	294 (240 – 347)	266 (212 – 317)
BioT (nmol/L)	7.1 (5.7 – 8.2)	6.2 (5.0 – 7.4)
SHBG (nmol/L)	31 (23 – 38)	42.7 (32 – 56)
AMS Score		
Total	N/A	29 (23 – 35)
Sexual	N/A	9 (6 - 12)
Somatic	N/A	13 (9 – 15)
Psychological	N/A	6 (5 – 9)
Comorbidities		
Current cigarette smokers	67 (11 %)	60 (9.6 %)
Diabetes Mellitus	24 (4.1 %)	44 (6.7 %)
Hyperlipidemia	48 (8.1 %)	137 (23 %)
Hypertension	87 (15 %)	181 (28 %)
Alcohol consumption (g/week)	39 (15 – 78)	40 (11– 78)
hsCRP	1.3 (0.7 – 2.3)	1.5 (0.8 – 2.7)
Physical Activity		
Inactive	40 (7 %)	73 (11 %)
Light	309 (54 %)	336 (53 %)
Moderate	203 (36%)	193 (30 %)
Vigorous	18 (3 %)	37 (6 %)

WHR, Waist-hip ratio; BMI, body mass index; TT, *total* testosterone; fT, *free* testosterone; BioT, *bioavailable* testosterone; SHBG, sex hormone-binding globulin; AMS, aging male symptom scale; hsCRP, high sensitive c-reactive protein.

### Cross-sectional associations between total testosterone levels and AMS scores at second visit

The cross-sectional differences in association between directly measured low *total* testosterone and normal testosterone concentration, and *total* AMS scores at the second visit was significant in Model 1 (Mean difference = 5.1, 95% CI: 1.7–8.5, p = 0.004). However, after adjustment for covariates, non-significant associations were observed (Model 2: Mean difference = 3.3, 95% CI: –0.1 to 6.7, p = 0.05; Model 3: Mean difference = 3.2, 95% CI: –0.4 to 6.8, p = 0.08; Model 4: Mean difference = 3.3, 95% CI: –0.3 to 6.9, p = 0.07) (**Table 2**). Furthermore, after adjusting for age and WHR, there was no statistically significant difference in AMS score between men with intermediate *total* testosterone with low calculated *free* testosterone vs. those with intermediate *total* and normal calculated *free* testosterone (mean AMS score 28.5 [95% CI 26.4–30.6] vs 29.0 [95% CI 26.9–31.0], respectively). Moreover, no significant associations were observed between calculated *bioavailable* testosterone and AMS score in linear or proportional odds logistic regression analyses, whether the AMS score was categorized as asymptomatic or symptomatic (mild/moderate/severe) (**Supplementary Table 2**). Likewise, there were no differences in symptom burden in men stratified by *total* testosterone levels (< 8 nmol/l, 8–11 nmol/l and ≥ 12 nmol/l) who had normal versus low calculated *free* testosterone (**Supplementary Figure 1**).

**Table 2:** Cross-sectional regression analyses between categorized directly measured *total* testosterone levels (< 8 nmol/l, 8–12 nmol/l and > 12 nmol/l) and AMS score at second visit.

Testosterone categories	< 8 nmol/L		8–12 nmol/L			> 12 nmol/L
Mean diff	95% CI	p	Mean diff	95% CI	p	Ref.
Unadjusted						
5.1	1.6, 8.5	<0.01	0.3	-1.6, 2.2	0.75	Ref.
Adjustment for age and WHR						
3.3	-0.1, 6.7	0.05	-0.2	-2.0, 1.7	0.87	Ref.
Adjustment for age, WHR, SHBG, cigarette smoking, alcohol consumption, LTPA						
3.2	-0.4, 6.8	0.08	0.6	-1.5, 2.7	0.55	Ref.
Adjustment for age, WHR, SHBG, cigarette smoking, alcohol consumption, LTPA, CRP, T2DM, hyperlipidemia, hypertension						
3.3	-0.3, 6.9	0.07	0.7	-1.4, 2.8	0.53	Ref.

WHR, Waist-hip ratio; SHBG, sex hormone-binding globulin; LTPA, leisure-time physical activity; T2DM, type 2 diabetes mellitus; Mean Diff, mean difference; 95% CI, 95% confidence interval).

### Cross-sectional associations between total testosterone levels and symptom domains at second visit

Subanalyses were done to further investigate the relationship between stratified *total* testosterone levels and AMS symptom domains. Statistically significant associations were found between low testosterone levels, compared to individuals with normal testosterone levels, and the sexual symptom domain in all models (Model 1; Mean difference = 2.4; 95% CI: 1.2, 3.7; p < 0.001, Model 2; Mean difference = 1.6; 95% CI: 0.4, 2.7; p = 0.008, Model 3; Mean difference = 1.6; 95% CI: 0.4, 2.9; p= 0.01, Model 4; Mean difference = 1.7; 95% CI: 0.4, 2.9; p = 0.01) (**Table 3**). Similarly, a statistically significant difference was shown for men with low testosterone levels and the somatic symptom domain, compared to men with normal levels in Model 1 (Mean difference = 2.3; 95% CI: 0.7, 4.0; p = 0.005) and in Model 2 (Mean difference = 1.7; 95% CI: 0.0, 3.3; p = 0.048), but not in Model 3 (Mean difference = 1.4; 95% CI: -0.3, 3.1; p = 0.1) and Model 4 (Mean difference = 1.4; 95% CI: -0.3, 3.2; p = 0.1) (**Table 3**). No statistically significant difference was observed between men with intermediate levels and normal levels for sexual and somatic symptom domains (**Table 3**). No significant associations were observed for the different testosterone groups with the AMS psychological symptom domain (**Table 3**).

**Table 3:** Cross-sectional regression analyses between categorized directly measured *total* testosterone levels (< 8 nmol/l, 8–12 nmol/l and > 12 nmol/l) and AMS domains (sexual, somatic and psychologic) at second visit.

Symptom domain	Testosterone groups	Unadjusted model	Model 1	Model 2	Model 3
		Mean diff (95% CI)	Mean diff (95% CI)	Mean diff (95% CI)	Mean diff (95% CI)
Sexual	≥ 12 nmol/L	Reference			
	8–11 nmol/L	0.2 (-0.6, 0.9)	0.1 (-0.5,0.8)	0.5 (-0.2,1.2)	0.5 (-0.2,1.2)
	< 8 nmol/L	2.4 (1.1, 3.7) **	1.6 (0.4, 2.7) **	1.6 (0.4,2.8) **	1.7 (0.4,2.9) **
Somatic	≥ 12 nmol/L	Reference			
	8–11 nmol/L	0.2 (-0.7,1.4)	-0.0 (-1.0,0.8)	0.2 (-0.7,1.2)	0.2 (-0.7,1.2)
	< 8 nmol/L	2.3 (0.7,4.0) **	1.7 (0.0,3.3)	1.4 (-0.3,3.1) *	1.6 (0.3,3.0) *
Psychological	≥ 12 nmol/L	Reference			
	8–11 nmol/L	0.2 (-0.7,0.4)	-0.3 (-0.9,0.3)	-0.2 (-0.9,0.5)	-0.2 (-0.9,0.4)
	< 8 nmol/L	0.2 (-0.7,1.1)	-0.006 (-0.9, 0.9)	0.2 (-0.8,1.3)	0.4 (-0.6,1.4)

**p<0.01, * p<0.05 WHR, Waist-hip ratio; SHBG, sex hormone-binding globulin; LTPA, leisure-time physical activity; T2DM, type 2 diabetes mellitus; Mean Diff, mean difference; 95% CI, 95% confidence interval. Adjustment was done for age and waist-to-hip ratio in Model 1, additional adjustment for SHBG, Cigarette smoking, Alcohol consumption and LPTA in Model 2, and final adjustment for T2DM, hyperlipidemia and hypertension in Model 3.

Due to differences in associations for men with low testosterone and sexual symptom domains, we investigated the relationship between low testosterone levels and individual symptom points in the sexual domain. Specific symptom items did not differ between men with intermediate and normal testosterone concentration (*data not shown)*. These analyses showed statistically significant associations between low testosterone concentrations and higher mean score for sexual symptoms compared to men with normal testosterone concentration (**Table 4**). The strongest association was related to self-reported *"decline in libido"* (mean difference 0.6, p = 0.002), followed by *"decrease in number of morning erection* ( 0.5, p = 0.014), and *"decrease in sexual performance"* (0.4, p = 0.032).

**Table 4:** Association between levels of directly measured *total* testosterone (TT) and symptoms in different items in AMS in sexual and somatic domains at second visit.

Domains/testosterone categories		TT <8nmol/L Mean score (95% CI)	TT > 12 nmol/L Mean score (95% CI)	Mean difference	P-value
Sexual symptoms	Decrease in number of morning erection	2.5 (2.1 – 2.9)	2.0 (1.9 – 2.1)	0.5	0.014
	Decrease in libido	2.5 (2.0 – 2.6)	1.9 (1.8 – 2.0)	0.6	0.002
	Decrease in sexual performance/erectile dysfunction	2.3 (2.0 – 2.5)	1.9 (1.8 – 2.0)	0.4	0.032
	Feeling of passing peak	2.3 (2.4 – 2.8)	2.3 (2.2 – 2.4)	0.	0.9
	Decreased in beard growth	1.0 (1.0 – 1.2)	1.1 (1.0 – 1.2)	- 0.1	0.10

Linear regression analyses between categorized *total* testosterone levels (< 8 nmol/L and > 12 nmol/L) and AMS individual sexual points. All analyses are fully adjusted.

### Sensitivity analyses: cross-sectional associations between recalculated testosterone and AMS scores at second visit

No statistically significant linear associations were observed between recalculated *total* testosterone and AMS score at second visit (Model 1: B = –0.1; 95% CI: –0.2, 0.1; p = 0.3; Model 2: B = –0.0; 95% CI: –0.2, 0.1; p = 0.90; Model 3: B = –0.02; 95% CI: –0.4, 0.0; p = 0.1; Model 4: B = –0.2; 95% CI: –0.4, 0.0; p = 0.1). However, there was evidence of differences between low testosterone levels (<8 nmol/L), compared to those with normal levels (≥12 nmol/L), and continuous AMS score in Model 1 (Mean difference = 3.7; 95% CI: 1.0, 6.4; p < 0.01), which was attenuated in Model 2 (Mean difference = 2.5; 95% CI: –0.2, 5.1; p = 0.07), but was significant in Models 3 and 4 (Model 3: Mean difference = 3.2; 95% CI: 0.3, 6.1; p = 0.03; Model 4: Mean difference = 3.7; 95% CI: 0.8, 6.6; p = 0.01) (**Supplementary Table 3**). No significant differences were found between intermediate and normal testosterone groups regarding AMS score (**Supplementary Table 3**). Similar results were found for the sexual domain and individual symptoms, while statistically significant associations were also seen for the somatic domain (**Supplementary Tables 4**, **5**).

### Longitudinal associations between testosterone and AMS scores

No statistically significant longitudinal associations were found between *total* or calculated *bioavailable* testosterone at first visit and AMS score at second visit, 10 years later, across all models (**Supplementary Tables 6**, **7**). Men with biochemical hypogonadism at first visit had higher AMS score at second visit compared to individuals with normal levels in the unadjusted analysis (Mean difference = 3.8; 95% CI: 0.5, 7.0, p = 0.03). No evidence in mean differences was observed in men with intermediate levels compared to men with normal *total* testosterone levels (Mean difference = 0.36; 95% CI: –1.4, 2.1; p = 0.69). Moreover, these associations attenuated and became non-significant after adjustment for age and waist-to-hip ratio (**Table 5**). Comparable findings were observed in proportional odds logistic regression analyses when AMS was analyzed categorically (asymptomatic, mild, moderate, severe), as well as for calculated *bioavailable* testosterone (*data not shown*). No changes in *total* testosterone concentration or significant symptom burden differ in men stratified by *total* testosterone levels (< 8 nmol/l, 8–11 nmol/l and ≥ 12 nmol/l) who had normal versus low calculated *free* testosterone (**Supplementary Figure 2**).

**Table 5:** Association between *total* testosterone at first visit and AMS score at second visit in longitudinal models.

Testosterone categories	< 8 nmol/L		8–12 nmol/L			> 12 nmol/L
Mean diff	95% CI	p	Mean diff	95% CI	P	Ref.
Unadjusted						
3.8	0.5, 7.1	0.03	0.36	-1.4, 2.1	0.69	Ref.
Adjustment for age and WHR						
2.0	-1.3, 5.3	0.23	-0.4	-2.1, 1.3	0.65	Ref.
Adjustment for age, WHR, SHBG, cigarette smoking, alcohol consumption, LTPA						
2.4	-1.2, 6.0	0.19	0.1	1.8,2.1	0.90	Ref.
Adjustment for age, WHR, SHBG, cigarette smoking, alcohol consumption, LTPA, CRP, T2DM, hyperlipidemia, hypertension						
2.3	-1.3,5.9	0.21	0.0	-2.0, 2.0	0.99	Ref.

Linear regression analyses were computed to investigate these associations. WHR, Waist-hip ratio; SHBG, sex hormone-binding globulin; LTPA, leisure-time physical activity; T2DM, type 2 diabetes mellitus; B, beta coefficient; 95% CI, 95% confidence interval.

## Discussion

Our findings indicate that men with low levels of endogenous *total* testosterone levels reported more severe symptoms on the AMS scale particularly regarding sexual health. To our knowledge, no prior study has demonstrated statistically significant associations between low endogenous *total* testosterone and the sexual domain in the AMS scale or investigated the trends longitudinally over a decade.

While testosterone prescriptions have expanded globally over the last two decades, clinical guidelines have remained largely unchanged, despite advancements in testosterone assays which still exhibit overestimation or variation in detecting low testosterone levels. In Sweden, and in Europe, a transition in testosterone immunoassay methodologies methodologies occurred, reflected notably in the Vara–Skövde cohort, where the Access Testosterone assay (Beckman Coulter) was replaced by the Elecsys Testosterone II assay (Roche Diagnostics; Testo II). This methodological shift resulted in systematically higher measured levels of *total* testosterone. Yet, the associations between low *total* testosterone levels and mainly sexual symptoms measured with the AMS scale aligns with the European guidelines reporting that men with sexual symptoms and testosterone levels below 8 nmol/L are diagnosed with late-onset hypogonadism (LOH) (4). Sexual symptoms (fewer morning erections, reduced libido and sexual performance) were significantly associated with low *total* testosterone levels in men compared with intermediate and normal *total* testosterone levels. This aligns with earlier studies by Wu et al. on which the European guidelines often refer to in their definition on LOH, defining it as men with three or more sexual symptoms (morning erection decline, decreased libido, erectile dysfunction) and *total* testosterone levels < 8 nmol/L, measured with gas chromatography-mass spectrometry (21). Although Wu et al. also classified symptomatic men with *total* testosterone <11 nmol/L and *free* testosterone <220 pmol/L (21) as hypogonadal, our study found no significant difference in AMS scores between men with intermediate *total* testosterone (8–12 nmol/L), whether their *free* testosterone was low or normal, and those with normal *total* testosterone levels. While these findings raise the question of whether men with intermediate *total* testosterone levels, regardless of their *free* testosterone status, should be considered for hypogonadal treatment when using immunoassay methods, the observed difference may be attributable to variations in androgen receptor polymorphism within the population (24).

While classical sexual symptoms are more common in younger men, older men often present with nonspecific complaints. Although sexual symptoms show the strongest association with biochemical hypogonadism, such symptoms in older men are frequently driven or amplified by comorbidities, particularly depression and cardiometabolic disorders (25, 26) This further underscores the diagnostic complexity of identifying late-onset hypogonadism in men. Yet, in this study, additional adjustments for comorbidities and medical use accentuated the relationship between low endogenous *total* testosterone and AMS scores in our study, suggesting negative confounding.

Due to change in immunoassay–based techniques, different relationships were observed between directly measured and recalculated *total* testosterone levels and symptoms of hypogonadism as assessed by the AMS score. Specifically, using directly measured *total* testosterone values at the second visit yielded a lower prevalence of biochemical hypogonadism and attenuated the statistical significance of the non-linear association between low *total* testosterone levels and AMS scores, relative to intermediate and normal levels, to borderline significance, indicative of reduced statistical power. However, the Testo II method appeared more specific, identifying fewer but more symptomatic men, observing similar mean differences compared to the recalculated method causing higher prevalence of biochemical hypogonadism. Although the Roche Testo II assay has been noted for potentially overestimating testosterone levels compared to other platforms, our comparative analysis suggests this does not compromise its clinical utility in this population. When we recalculated testosterone values to harmonize them with the Beckman-Coulter Access method, the number of men categorized as biochemically hypogonadal (< 8 nmol/L) increased from 32 to 51. However, the mean symptom burden, most notably in the sexual domain, remained remarkably consistent between the two groups.

These findings indicate that while the direct Testo II measurement may be more conservative in its classification, it successfully identifies the most symptomatic individuals. The stability of the symptom scores across both measurement approaches suggests that the observed associations between low *total* testosterone and AMS scores are robust and not merely an artifact of the specific assay platform used. Furthermore, we observed robust longitudinal tracking of total testosterone levels over the 10-year study interval (**Figure 1**), indicating intra-individual biological stability over a decade. This correlation underscores that despite the systemic shift introduced by the laboratory assay platform migration, the ranking of individuals’ testosterone levels remained relative preserved over time.

The implications of this technique transition have previously been examined by Owen et al., who compared both assays to the liquid chromatography–tandem mass spectrometry (LC-MS/MS) (15). While the Testo II assay demonstrated improved sensitivity for detecting testosterone levels in women and children, both methods have shown higher agreement with LC-MS/MS for identifying hypogonadal thresholds in men. Nonetheless, in our study, the coefficient of variation (CV) was higher for the Beckman Coulter assay than for the Testo II.

When we applied recalculated testosterone values, the prevalence of hypogonadal levels rose and significant associations between recalculated *total* testosterone levels and somatic complaints were observed, specifically physical exhaustion/lack in vitality, while psychological symptoms remained unrelated with both directly measured and recalculated methods, similarly as earlier findings (21). This suggests that although hypogonadal men may experience psychological symptoms, using nonspecific symptoms in questionnaires or as a screening criterion, these symptoms could inflate symptom scores and result in unnecessary blood testing and over screening. While testosterone is essential for muscle maintenance, its weak associations with somatovegetative symptoms suggest that these complaints, or the questionnaire items used to assess them, may not specifically reflect hypogonadism. Future research should undertake qualitative work (e.g., semi-structured interviews) to refine symptom constructs prior to quantitative assessment.

Previous studies have reported only weak or non-significant relationships between circulating testosterone levels and AMS scores (27–30). Several factors may underlie these discrepancies, including the non-specific nature of many AMS symptoms, potential contributions from other hormonal systems, symptoms related with aging and potentially attributable to assay methodologies that potentially could yield systematically elevated testosterone measurements, introducing method-dependent measurement bias (10, 29, 31). Although the AMS questionnaire may aid in monitoring treatment response (32) or characterizing symptoms in men with established LOH, its limited specificity makes it unsuitable as a diagnostical tool. An updated version focusing on sexual and somatic domains, with psychological items used to capture alternative or contributing causes, could improve its clinical utility. However, even sexual and somatic symptoms may arise from other conditions such as cardiovascular disorders, which should be considered during evaluation (25).

Finally, we did not observe any longitudinal associations between baseline testosterone levels and AMS score 10 years later, after adjusting for age and waist–hip ratio, suggesting that age and adiposity could have a greater influence on long-term symptoms than initial testosterone levels.

### Strengths and limitations

Strengths of this study include a fairly large population-based sample with longitudinal data from two visits. Moreover, a further strength is the detailed collection of information and blood samples, nearly identical protocols between the two visits and using validated instruments. However, there are some limitations to this study. First, two different immunoassays were used to assess testosterone levels in men. Earlier studies have shown that immunoassay methods are considered less reliable compared to mass-spectrometry, both in intermediate ranges but also a difference in correlations between techniques with testosterone levels <8nmol/l (33, 34). Because the Roche platform systematically overestimates total testosterone by approximately 11% compared to the Beckman Access Testo assay, a cross-temporal correction factor of 0.89 was applied. However, as a single linear scaling factor cannot correct for platform-specific non-linearity at the lower limits, our primary clinical threshold remains inherently assay-dependent. To maintain full transparency, we have presented the raw, platform-specific values as our primary analysis and reframed the recalculated model strictly as a sensitivity analysis. Yet, studies have also shown that immunoassays can fairly equate to mass spectrometry in distinguishing between eugonadal and hypogonadal testosterone levels in men, particularly when interpreted alongside clinical indicators of androgen deficiency (34). This recommendation is likewise articulated in the guidelines published by the European Academy of Endocrinology (4). Although the use of two different assay methods might be considered a limitation, our analysis demonstrates that both approaches yield highly consistent results regarding symptom burden. This consistency highlights the robustness of the association between testosterone and AMS scores, regardless of the platform used. Notably, utilizing uncorrected Roche values weakens the total testosterone-AMS association to borderline significance (p =.07). This behavior is epidemiologically consistent with differential misclassification; because the Roche assay systematically skews higher, truly hypogonadal, symptomatic men are misclassified into the eugonadal reference group, thereby diluting the case cohort. Applying the 0.89 correction serves as a conservative sensitivity check to mitigate this platform drift. Furthermore, the transition to the Testo II assay reflects the current diagnostic standard in Europe, while it may require a nuanced interpretation of cut-off values, its improved performance in the lower ranges makes it a more reliable tool for detecting clinically relevant hypogonadism in a modern population. Furthermore, while some baseline differences existed between follow-up attendees and non-attendees (**Supplementary Table 8**), these were accounted for in the adjusted models. Second, a limitation in this study is the use of calculated bioactive testosterone levels according to Vermeulen, which have been shown to overestimate hormone levels (35). Third, the small number of biochemically hypogonadal men after recalibration limits statistical power and increases the risk of type II error, complicated by already method-dependent measurement bias. Finally, the observational, cross-sectional nature of these analyses precludes causal inference and leaves the findings vulnerable to residual confounding, selection bias, and temporal ambiguity. Regarding selection bias, participants with more severe somatic or psychological symptoms may have been less likely to participate at the second time point, which could influence and potentially underestimate the observed longitudinal associations. However, empirical comparison of baseline data between follow-up participants and non-participants (**Supplementary Table 8**) demonstrated a remarkably high degree of homogeneity across testosterone and SHBG levels, BMI, abdominal adiposity, lifestyle habits, inflammation, and metabolic comorbidities. The only notable divergence was a slightly higher baseline age among attendees (median 48 vs. 44 years), which was controlled for in all adjusted models. Furthermore, the observed associations between AMS scores and cross-sectional testosterone levels may reflect reverse causation. Rather than low hormone levels directionally driving these clinical manifestations, severe symptoms, along with underlying chronic health conditions or systemic stress, can suppress the hypothalamic-pituitary-gonadal axis, thereby lowering endogenous testosterone production. Prospective, longitudinal studies are essential to clarify the directionality of these relationships.

## Conclusion

Low endogenous testosterone concentrations were primarily associated with sexual symptoms defined by AMS questionnaire, while somatic and psychological symptoms were unrelated. Although the transition between measurements resulted in a numerical shift in testosterone values, the clinical association remained consistent across both methods, demonstrating that the modern assay identifies the most symptomatic individuals despite categorizing fewer men as biochemically low.

## Data Availability

The datasets presented in this article are not readily available because the datasets analyzed during the current study are not publicly available. The data are, however, available from the corresponding author upon reasonable request and with the permission of the relevant institutional review board or data governing committee. Requests to access the datasets should be directed to Amar Osmancevic, amar.osmancevic@gu.se.
