# Supplementary material for: Testosterone levels and symptoms of hypogonadism in Swedish men: a prospective study from the Vara – Skövde cohort
Source: Front Endocrinol (Lausanne). 2026 Jul 17;17:1882696. doi: 10.3389/fendo.2026.1882696 (PMC13423733; doi:10.3389/fendo.2026.1882696)

Supplementary Table 1. Mean Differences in AMS scores (Total, Sexual, Somatic, and Psychological Domains) across directly measured and recalculated total testosterone categories (<8 nmol/L, 8–12 nmol/L, >12 nmol/L) at second visit.

| Testosterone group (nmol/l) | Measurement Type | N | \| AMS Total (Mean [SD]) \| \| --- \| | \| AMS Sexual (Mean [SD]) \| \| --- \| | \| AMS Somatic (Mean [SD]) \| \| --- \| | \| AMS Psychological (Mean [SD]) \| \| --- \| |
| --- | --- | --- | --- | --- | --- | --- | --- | --- | --- | --- |
| < 8 nmol/l | Directly measured | 32 | 34 (± 11) | 11 (± 4) | 15 (± 5) | 8 (± 3) |
|  | Recalculated | 51 | 32 (± 11) | 11 (± 4) | 14 (± 5) | 8 (± 3) |
|  | | | | | | |
| 8 - 12 nmol/l | Directly measured | 117 | 30 (± 9) | 9 (±4) | 13 (± 4) | 8 (± 3) |
|  | Recalculated | 145 | 29 (± 9) | 9 (±3) | 13 (± 4) | 8 (± 3) |
|  | | | | | | |
| >12 nmol/l | Directly measured | 443 | 29 (± 9) | 9 (± 3) | 13 (± 4) | 8 (± 3) |
|  | Recalculated | 391 | 29 (± 9) | 9 (± 3) | 13 (± 4) | 8 (± 3) |

Standard Deviation (SD), Aging Male Symptom (AMS) scale.

Supplementary Table 2. Cross-sectional regression analyses between calculated bioavailable testosterone and AMS score in linear and proportional odds logistic regression analyses.

| Analysis Type | Model | Estimate | 95% CI | p-value |
| --- | --- | --- | --- | --- |
| Linear Regression | Model 1 | -0.788 | (-1.169, -0.407) | < 0.001 |
| Total AMS Score | Model 2 | -0.196 | (-0.613, 0.221) | 0.357 |
|  | Model 3 | -0.294 | (-0.727, 0.139) | 0.182 |
|  | Model 4 | -0.285 | (-0.717, 0.146) | 0.195 |
|  | | | | |
| Logistic Regression | Model | OR | 95% CI | p-value |
|  | Model 1 | 0.861 | (0.793, 0.934) | < 0.001 |
| AMS Groups | Model 2 | 0.983 | (0.897, 1.077) | 0.710 |
|  | Model 3 | 0.965 | (0.876, 1.063) | 0.470 |
|  | Model 4 | 0.966 | (0.877, 1.065) | 0.489 |

Supplementary Table 3. Cross-sectional regression analyses between categorized recalculated total testosterone levels (< 8 nmol/l, 8-12 nmol/l and > 12 nmol/l) and AMS score at second visit.

| Testosterone concentrations | < 8 nmol/L | | 8-12 nmol/L | | | > 12 nmol/L |
| --- | --- | --- | --- | --- | --- | --- |
| Mean diff | 95 % CI | p | Mean diff | 95 % CI | p | Ref. |
| Unadjusted | | | | | | |
| 3.7 | 1.0, 6.3 | <0.01 | -0.3 | -2.4, 1.5 | 0.75 | Ref. |
| Adjustment for age and WHR | | | | | | |
| 2.5 | -0.2, 5.1 | 0.07 | -0.3 | -2.0, 1.5 | 0.75 | Ref. |
| Adjustment for age, WHR, SHBG, cigarette smoking, alcohol consumption, LTPA | | | | | | |
| 3.2 | 0.3, 6.1 | 0.03 | -0.4 | -2.3, 1.6 | 0.70 | Ref. |
| Adjustment for age, WHR, SHBG, cigarette smoking, alcohol consumption, LTPA, CRP, T2DM, hyperlipidemia, hypertension | | | | | | |
| 3.7 | 0.8, 6.6 | 0.01 | -0.4 | -2.4, 1.5 | 0.66 | Ref. |

Waist-hip ratio (WHR), sex hormone-binding globulin (SHBG), leisure-time physical activity (LTPA), type 2 diabetes mellitus (T2DM) mean difference (Mean Diff), 95% confidence interval (95 % CI).

Supplementary Table 4. Cross-sectional regression analyses between categorized recalculated total testosterone levels (< 8 nmol/l, 8-12 nmol/l and > 12 nmol/l) and AMS domains (sexual, somatic and psychologic) at second visit.

| Symptom domain | Testosterone groups | Unadjusted model | Model 1 | Model 2 | | Model 3 |
| --- | --- | --- | --- | --- | --- | --- |
|  |  | Mean diff (95 % CI) | Mean diff (95 % CI) | | Mean diff (95 % CI) | Mean diff (95 % CI) |
| Sexual | ≥ 12 nmol/L | Reference | | | | |
|  | 8-11 nmol/L | -0.1 (-0.8, 0.6) | -0.2 (-0.8,0.4) | | 0.1 (-0.6,0.7) | 0.04 (-0.6,0.7) |
|  | < 8 nmol/L | 1.7 (0.6, 2.7) ** | 1.2 (0.3, 2.1) ** | | 1.5 (0.5,2.5) ** | 1.7 (0.7,2.7) ** |
| Somatic | ≥ 12 nmol/L | Reference | | | | |
|  | 8-11 nmol/L | -0.05 (-0.89,0.8) | -0.3 (-1.1,0.6) | | 0.2 (-1.1,0.7) | -0.2 (-1.1,0.7) |
|  | < 8 nmol/L | 1.8 (0.5,3.1) ** | 1.3 (-0.0,2.6) | | 1.4 (0.0,2.8) * | 1.6 (0.3,3.0) * |
| Psychological | ≥ 12 nmol/L | Reference | | | | |
|  | 8-11 nmol/L | 0.2 (-0.7,0.4) | -0.3 (-0.9,0.3) | | -0.2 (-0.9,0.5) | -0.2 (-0.9,0.4) |
|  | < 8 nmol/L | 0.2 (-0.7,1.1) | -0.006 (-0.9, 0.9) | | 0.2 (-0.8,1.3) | 0.4 (-0.6,1.4) |

**p<0.01, * p<0.05 Waist-hip ratio (WHR), sex hormone-binding globulin (SHBG), leisure-time physical activity (LTPA), type 2 diabetes mellitus (T2DM), mean difference (Mean Diff), 95% confidence interval (95 % CI). Adjustment was done for age and waist-to-hip ratio in Model 1, additional adjustment for SHBG, Cigarette smoking, Alcohol consumption and LPTA in Model 2, and final adjustment for T2DM, hyperlipidemia and hypertension in Model 3.

| Supplementary Table 5. Association between levels of re-calculated total testosterone (TT) and symptoms in different items in AMS in sexual and somatic domains at second visit. | | | | | |
| --- | --- | --- | --- | --- | --- |
| Domains/Testosterone Categories | | TT <8nmol/L  Mean score (95 % CI) | TT > 12 nmol/L  Mean score (95 % CI) | Mean Difference | p-value |
| Sexual symptoms | Decrease in number of morning erection | 2.5  (2.1 – 2.9) | 2.0  (1.9 – 2.1) | 0.5 | <0.001 |
|  | Decrease in libido | 2.5  (2.0 – 2.6) | 1.9  (1.8 – 2.0) | 0.6 | <0.001 |
|  | Decrease in sexual performance/erectile dysfunction | 2.3  (2.0 – 2.5) | 1.9  (1.8 – 2.0) | 0.4 | <0.001 |
|  | Feeling of passing peak | 2.6  (2.4 – 2.8) | 2.3  (2.2 – 2.4) | 0.3 | 0.04 |
|  | Decreased in beard growth | 1.1  (1.0 – 1.2) | 1.1  (1.0 – 1.2) | 0.0 | 0.5 |
|  | | | | | |
| Somatic Symptoms | Decline in your feeling of general well-being | 2.6 (1.8 – 2.8) | 2.3 (1.7 – 2.5) | 0.3 | 0.06 |
|  | Joint pain and muscular ache | 2.6 (2.4 – 2.9) | 2.4 (2.3 – 2.5) | 0.2 | 0.1 |
|  | Excessive sweating | 1.5 (1.3 – 1.6) | 1.3 (1.2 – 1.3) | 0.2 | 0.05 |
|  | Sleep problems | 2.0 (1.5 – 2.1) | 1.8 (1.7 – 1.9) | 0.2 | 0.2 |
|  | Increased need for sleep, often feeling tired | 2.0 (1.8 – 2.3) | 1.8 (1.7 – 2.0) | 0.2 | 0.2 |
|  | Physical exhaustion / lacking vitality | 2.2 (1.8 – 2.3) | 1.8 (1.7 – 1.9) | 0.4 | 0.007 |
|  | Decrease in muscular strength | 2.1 (1.8 – 2.2) | 1.9 (1.7 – 1.9) | 0.2 | 0.09 |

Linear regression analyses between categorized total testosterone levels (< 8 nmol/l, 8-11 nmol/l and ≥ 12 nmol/l) and AMS individual sexual and somatovegetative points. All analyses were fully adjusted.

Supplementary Table 6. Longitudinal associations between total testosterone at baseline and AMS at follow-up.

| Model | Coefficient (β) | 95% CI | p-value |
| --- | --- | --- | --- |
| Model 1 | -0.073 | (-0.239, 0.092) | 0.385 |
| Model 2 | 0.042 | (-0.125, 0.209) | 0.621 |
| Model 3 | -0.006 | (-0.230, 0.218) | 0.958 |
| Model 4 | -0.005 | (-0.230, 0.220) | 0.966 |

Unadjusted associations in Model 1, further adjustment for age and waist-to-hip ratio in Model 2, additional adjustment for SHBG, Cigarette smoking, Alcohol consumption and LPTA in Model 3, and final adjustment for T2DM, hyperlipidemia and hypertension in Model 4.

Supplementary Table 7. Longitudinal associations between calculated bioavailable testosterone at baseline and AMS at follow-up.

| Model | Coefficient (β) | 95% CI | p-value |
| --- | --- | --- | --- |
| Model 1 | -0.519 | (-0.887, -0.150) | 0.006 |
| Model 2 | 0.046 | (-0.352, 0.444) | 0.820 |
| Model 3 | 0.047 | (-0.359, 0.454) | 0.819 |
| Model 4 | 0.049 | (-0.359, 0.458) | 0.813 |

Unadjusted associations in Model 1, further adjustment for age and waist-to-hip ratio in Model 2, additional adjustment for SHBG, Cigarette smoking, Alcohol consumption and LPTA in Model 3, and final adjustment for T2DM, hyperlipidemia and hypertension in Model 4.

Supplementary Table 8. Sensitivity analyses comparing those who attended follow up vs those who did not attend follow-up.

| **Characteristic** | 0  N = 743^1^ | 1  attended follow-up  N = 657^1^ | p-value^2^ |
| --- | --- | --- | --- |
| Age | 44 (37, 50) | 48 (40, 59) | <0.001 |
| BMI | 26.3 (24.3, 28.9) | 26.4 (24.6, 28.8) | 0.6 |
| Waist-to-hip ratio | 0.94 (0.90, 0.99) | 0.94 (0.90, 0.98) | 0.6 |
| Leisure Time Physical Activity |  |  | 0.7 |
| 1 | 55 (7.6%) | 52 (8.2%) |  |
| 2 | 375 (52%) | 338 (53%) |  |
| 3 | 256 (36%) | 224 (35%) |  |
| 4 | 33 (4.6%) | 21 (3.3%) |  |
| Alcohol Consumption | 37 (10, 81) | 35 (14, 77) | 0.9 |
| Diabetes Mellitus Type 2 | 54 (7.3%) | 35 (5.3%) | 0.14 |
| Triglycerides | 1.23 (0.87, 1.74) | 1.22 (0.89, 1.73) | 0.7 |
| Testosterone nmol/l | 13.9 (11.5, 16.8) | 14.0 (11.3, 17.1) | >0.9 |
| SHBG | 30 (23, 40) | 31 (23, 39) | 0.4 |
| CRP | 1.26 (0.73, 2.55) | 1.26 (0.74, 2.33) | 0.5 |
| Hypertension | 107 (14%) | 102 (16%) | 0.6 |

^1^Median (Q1, Q3); n (%)

^2^Wilcoxon rank sum test; Pearson's Chi-squared test

Supplementary Table 9. Sensitivity analyses comparing those with missing values in AMS with those with missing data on AMS.

| **Characteristic** | 0 (missing data on AMS) N = 67^1^ | 1  N = 590^1^ | p-value^2^ |
| --- | --- | --- | --- |
| ageyr | 57 (45, 67) | 47 (39, 57) | <0.001 |
| bmi | 27.4 (25.0, 29.0) | 26.4 (24.5, 28.8) | 0.3 |
| whr | 0.96 (0.89, 0.99) | 0.94 (0.90, 0.98) | 0.3 |
| Leisure Time Physical Activity |  |  | 0.018 |
| 1 | 12 (18%) | 40 (7.0%) |  |
| 2 | 29 (45%) | 309 (54%) |  |
| 3 | 21 (32%) | 203 (36%) |  |
| 4 | 3 (4.6%) | 18 (3.2%) |  |
| Unknown | 2 | 20 |  |
| Smoking |  |  | 0.011 |
| 1 | 31 (46%) | 286 (48%) |  |
| 2 | 19 (28%) | 230 (39%) |  |
| 3 | 17 (25%) | 74 (13%) |  |
| Alcohol Consumption | 15 (0, 34) | 39 (15, 78) | <0.001 |
| Unknown | 1 | 17 |  |
| SHBG | 34 (26, 44) | 31 (23, 38) | 0.023 |
| Unknown | 0 | 7 |  |
| Hyperlipidemia | 10 (15%) | 46 (7.8%) | 0.044 |
| Unknown | 1 | 3 |  |
| Diabetes Mellitus Typ 2 |  |  | <0.001 |
| 0 ( no diabetes) | 56 (84%) | 566 (96%) |  |
| 1 (diabetes) | 11 (16%) | 24 (4.1%) |  |
| Hypertension | 15 (22%) | 87 (15%) | 0.10 |
| ^1^Median (Q1, Q3); n (%) | | | |
| ^2^Wilcoxon rank sum test; Fisher's exact test; Pearson's Chi-squared test | | | |

Supplementary Figure 1. Comparison of symptom burden in men stratified by total testosterone levels (< 8 nmol/L, 8-12 nmol/L and > 12 nmol/L) and categorized by normal versus low calculated free testosterone in fully adjusted model. A subgroup characterized by low total testosterone and high free testosterone is not included because this phenotype is a biological and mathematical rarity due to the calculated nature of free testosterone, and no participants fell into this category within our baseline cohort distribution.


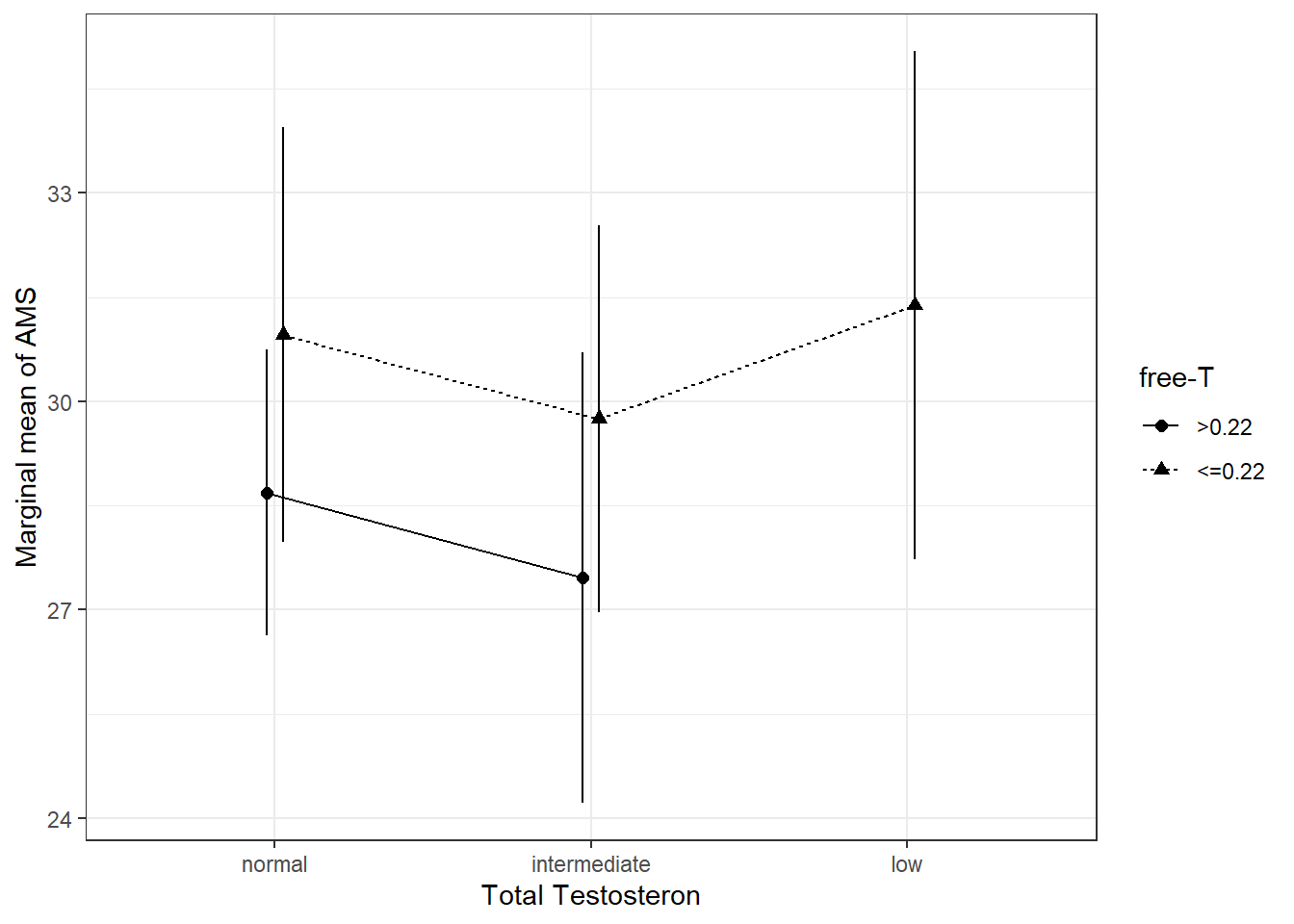


Supplementary Figure 2. Comparison of symptom burden in men stratified by total testosterone levels (< 8 nmol/L, 8-12 nmol/L and > 12 nmol/L) at baseline and categorized by normal versus low calculated free testosterone in fully adjusted model.


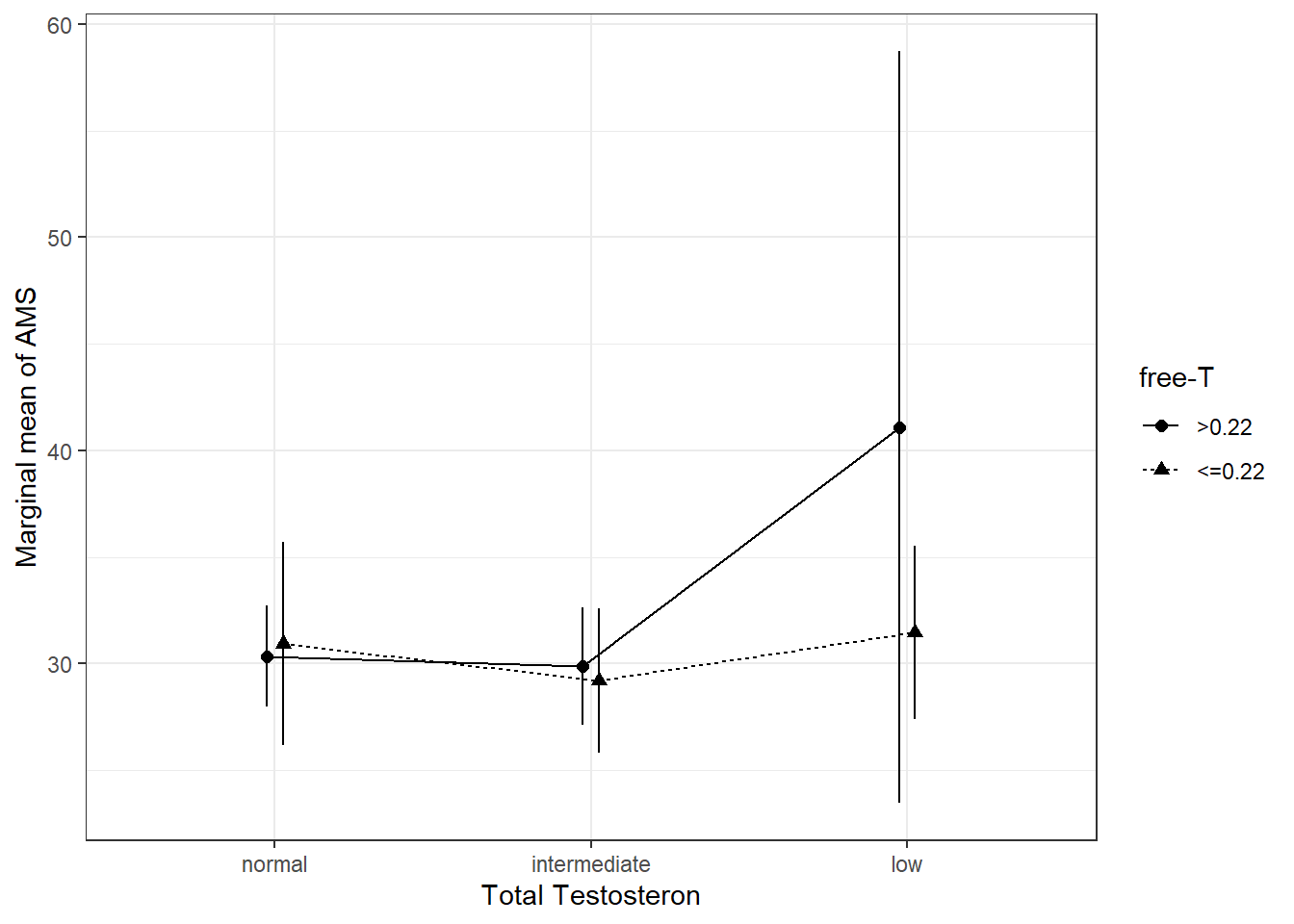

Supplement: Supplementary file 1 [file DataSheet1.docx]
